# Changing the Name of Schizophrenia: Patient Perspectives and Implications for DSM-V

**DOI:** 10.1371/journal.pone.0055998

**Published:** 2013-02-14

**Authors:** Constantin Tranulis, Tania Lecomte, Bassam El-Khoury, Anaïs Lavarenne, Daniel Brodeur-Côté

**Affiliations:** 1 Department of Psychiatry, University of Montreal, Montreal, Québec, Canada and Louis-H. Lafontaine Hospital, Montreal, Quebec, Canada; 2 Department of Psychology, University of Montreal, Montreal, Québec, Canada; Baylor College of Medicine, United States of America

## Abstract

**Introduction:**

The diagnosis of schizophrenia is increasingly contested by researchers, clinicians, patients and family members. Preeminent researchers proposed its replacement with the salience syndrome concept, arguing for increased validity and less stigmatizing potential. This is the first study exploring the effects on stigma of this nosological proposal.

**Methods:**

Two studies were conducted: one with 161 undergraduate students regarding their stigmatizing attitudes linked to the label of schizophrenia or salience syndrome, the other involved in-depth qualitative interviews with 19 participants treated in a first episode psychosis program. The interviews explored the subjective validity, acceptability and effects on stigma of a diagnosis of schizophrenia or salience syndrome.

**Results:**

Overall, no significant differences were found between labels in study 1. For study 2, the majority of participants preferred a diagnosis of salience syndrome, considering it less stigmatizing mostly because of its novelty and the concealing potential of the new diagnostic entity, though many found it hard to relate to and somewhat difficult to understand.

**Discussion:**

Our results suggest that the label change does not impact the stigmatizing potential for individuals who are not familiar with mental illness - they appear to base their attitudes on descriptions rather than the label alone. For those suffering from mental illness, a name change for schizophrenia to “salience syndrome” might offer only a temporary relief from stigma. Claims of de-stigmatizing effects should be grounded in sound scientific models of stigma and ideally in empirical data.

## Introduction

More than 100 years after its introduction [1], the diagnosis of schizophrenia continues to create controversies and intense debates (see for example the British Medical Journal editorials [2], [3] and the ensuing lively debate [4]). In resonance with scientific critiques of the validity of this nosographic concept, it is well established that the label of “schizophrenia” can be profoundly invalidating and stigmatizing. Stigma can be understood as a process with six dimensions: labeling, stereotyping, separation, emotional reaction, discrimination and power differential [5]. Consequently, several authors have proposed alternative diagnostic entities (e.g. the “deconstructing psychosis” conference [6], in preparation of the new version of the Diagnostic and Statistical Manual of Mental Disorders (DSM) [7]). Patient and family groups suggested renaming the condition and proposed a plethora of alternative labels [8]. However, the term schizophrenia remains the principal diagnostic category of the International Classification of Diseases and Diagnostic Statistical Manual of mental disorders and it is widely used in clinical, research and popular contexts, with the notable exception of Japan. Indeed, the Japanese Society of Psychiatry and Neurology changed in 2002 the diagnostic label of “Seishin Bunretsu Byo” (“mind-split-disease”, equivalent to schizophrenia) for “Togo Shitcho Sho” (integration disorder). Increased acceptability by psychiatrists and higher rates of informing the patients and families were reported after the introduction of the new term [9]. Using an Implicit Attitudinal Test, Takahashi and colleagues explored the automatic associations between the old and new schizophrenia labels and negative stereotypes (in this case, violence). In a sample of 68 young Japanese students, they observed a weaker association with the term “criminal” for the new diagnostic term [10], suggesting that the new diagnostic term “holds promise for tempering the negative bias toward the disorder in Japan”. In another empirical study, 241 British medical students rated their attitudes to five alternative labels (sensitivity-, drug-, anxiety-, traumatic psychoses and schizophrenia). These hypothetical changes in terminologies also showed fewer explicit negative attitudes in comparison to schizophrenia [11]. However, these reports of attitudinal changes are prone to several biases, in particular social desirability [5]. A wealth of sociological research showed convincingly that terminology changes do not influence durably stigma and its manifestations (i.e. discrimination), with most researchers proposing a multi-modal approach, such as the Protest-Educate-Contact model [12]. Indeed, the theoretical model of stigma predicts only a limited link between attitudinal changes and the core dimensions of stigma and discrimination (i.e. the attitudes/stereotype is only one of the six dimensions).

Not only is there a paucity of studies of stigma and label changes of schizophrenia, but the perspective of the principal stakeholders, the patients themselves, is essentially absent from these scientific inquiries.

In this context, several proposals to change the name of schizophrenia have been made. Shitij Kapur [13], [14] and Jim van Os [15] proposed to replace the psychosis spectrum disorders (including schizophrenia) with “(abnormal) salience syndrome” in DSM-V. This was arguably the most popular alternative for schizophrenia among the scientific community. Salience refers to how internal and external stimuli are consciously experienced and how, unwilled or overinclusive attention to some stimuli can become perplexing and foster a search for explanations which are later recognized as delusional [14]. The abnormal salience is postulated to be the core cognitive problem experienced by individuals suffering from psychosis. The authors suggest that salience syndrome has better scientific validity, better clinical validity (in the sense that it is closer to the lived experience of patients) and would be less stigmatizing than the label of schizophrenia. While these hypotheses sound convincing, they are in need of empirical validation. In order to explore these questions, we wished to investigate both the attitudinal impact of the label in an often-studied convenience sample (i.e. university students), as well as to explore scientifically whether 1) the “salience syndrome” is perceived as useful by the patients and 2) in which way it might influence expected stigma, including self-stigma. Specifically, we wished to study these last questions with an early psychosis sample. Early psychosis is a period of intense negotiation of meaning for patients, which includes reflecting about diagnoses. Because most individuals treated for early psychosis have not yet received or accepted a diagnosis of schizophrenia, they might be less likely to be biased or to show rigidity in their evaluations of new nosological entities. They are also likely to have fresh memories of their experiences of psychosis and to be interested to reflect about the most appropriate diagnostic label.

## Methods

The study was conducted in two parts. Both studies were approved by the University of Montreal’s Institutional Review Board, and the Hôpital Louis-H. Lafontaine’s Ethic’s and Scientific review boards, and all participants gave written informed consent to participate in the study.

### Study 1

#### Participants

Study 1 involved 161 undergraduates from various programs studying at the University of Montreal. The average age was 24 (S.D. = 5.8), 62% were women, and the most represented programs were psychology (20%) and biological sciences (20%).

#### Procedure

Following approval from the University of Montreal’s Institutional Review Board, students were approached in their classrooms and asked to read a vignette describing a young man named Nathan who appeared to have changed recently, becoming more and more withdrawn, missing classes, with a disheveled presentation, and mentioning feeling persecuted by teachers. Half the students received version A of the vignette, which mentioned that Nathan was given the diagnosis of salience syndrome, and described the symptoms as feelings of distress and increased importance given to internal and external stimuli, followed by unusual experiences and irrational thoughts. The other half was given version B, which offered the exact same description but mentioned that Nathan had a diagnosis of schizophrenia. In both versions the psychiatrist prescribed antipsychotic medication, as well as psychoeducation and psychotherapy.

#### Measure

Other than their age, study program, gender and email (if they wished to participate in a raffle), following each version, the students answered the following five questions, on a five point Likert scale ranging from very unlikely (−2), to neutral (0), and to very likely (+2).):

*Please underline the answer which best reflects your views:*

1. *Do you think that this would damage Nathan’s career?*
2. *I would be comfortable if Nathan was my colleague at work?*
3. *I would be comfortable about inviting Nathan to a dinner party?*
4. *How likely do you think it would be for Nathan’s girlfriend to leave him?*
5. *How likely do you think it would be for Nathan to get in trouble with the law?*

### Study 2

#### Participants

Twenty patients treated in a first episode psychosis (FEP) clinic were approached to participate in the study when the treating clinician considered them clinically stable and competent to offer informed consent. One participant appeared very guarded and gave short or evasive answers to all the questions (e.g. “I don’t have a story to tell” was the longest sentence, when asked for the general illness narrative). After revision of the case, we decided to exclude this participant, as the quality and the reliability of the interview were sub-optimal. For the final sample of 19 participants, the average age was 34, 9 were women and the average psychiatric follow-up was of 3 years (range 0 to 8 years).

#### Procedure

Two doctoral students in psychology were hired as interviewers. After explaining the study and obtaining written informed consent, they also stressed the confidential nature of the study, notably in relationship with the treating team. They positioned themselves as unrelated to the clinical staff, neutral and interested in the subjective experiences of the research participants. The interviewers were trained to use the same language and concepts as the participants.

#### Measure

The information collected in study 2 stemmed from three sources: an in-depth semi-structured interview, self-report questionnaires and chart review.

The interview contained three parts. Firstly, a brief illness narrative was obtained from each participant. This had a triple goal: develop rapport with the participants, qualitatively understand their view of their mental health problem and provide background information for a more valid interpretation of the responses generated later. Then, two clinical vignettes were presented in random order, each depicting a gender- and age- matched person suffering either from schizophrenia or salience syndrome (cf. appendix S1). Thirdly, participants were asked to choose which of the two descriptions was more acceptable and which fitted better with their own experience of illness. They were encouraged to elaborate on similarities and disparities between their subjective experiences and the clinical vignettes. The participants were specifically asked to reflect on which diagnostic had less stigmatizing potential and why.

Sociodemographic and illness information were obtained via self-report and chart review, whereas the experience of self-stigma was explored with the Internalized Stigma of Mental illness Scale (ISMIS; [16] - a self-report questionnaire of 29 items rated on a 4 point Likert scale). The scale offers four subscales: alienation, stereotype endorsement, discrimination experience, and social withdrawal. Further qualitative exploration of stigma was then conducted by asking questions based on the six dimensional model of stigma (labeling, stereotyping, separation in us and them, emotional reactions, status loss and discrimination, and power imbalances) [17]. The interviews were taped and later transcribed. After each interview, a brief ethnographic note was completed in order to complete the audio recording with non-verbal information about the participant and the context of the interview, including evaluations of collaboration. Concerns over the reliability of the answers were noted when present.

### Analyses

For Study 1 and 2, descriptive, t-tests or Anovas were conducted using SPSS v19. For Study 2, involving the qualitative analyses, the transcribed interviews were analyzed with Dedoose 3.3.66. We developed a coding structure based on the six dimensions of stigma, the preferred label and the acceptability of diagnosis. Four additional codes were added in an iterative manner during the analysis when new content was present: self-reported insight, concealment, recovery potential and experiential fit (i.e. comments about how the two vignettes are related to subjective experiences). Qualitative data analysis was performed by CT and was shared with the co-investigator (TL) and the two interviewers (BK, AL) for feedback and adjustments.

## Results

### Study 1

Table S1 shows the means and standard deviations for the sample as a whole for both versions (A: salience, and B: schizophrenia) for the student sample. As can be seen, similar stigmatizing attitudes were found for each question for both versions, with the domains of social distance at work (comfort with colleague) and in social interactions (dinner party) being most likely affected by the condition depicted in the vignette. Post-hoc analyses were then conducted in order to compare the attitudes of students who might *a priori* know more about mental illness (i.e. those in psychology) from the students in the other biggest program in our sample (biology). Student’s t-tests revealed that for the total score, individuals in psychology showed a trend towards having a more positive attitude in terms of social distance (both at work and socially, questions 2 and 3) for both diagnostic terms; this difference was significant only for social distance in a social situation (question 3) when presented with the diagnosis of salience syndrome (t (38) = −2.09, p<0.05).

### Study 2

In the 19 participants of study 2, cooperation and reliability were considered good to excellent. The interviews lasted between 18 and 59 minutes, with an average of 28 minutes.

Seven out of 19 participants self-reported a diagnosis of schizophrenia, two identified a diagnosis of psychosis and two of schizo-affective disorder, four reported a non-psychotic diagnosis (anxiety, depression, hypnosis proneness) and, four denied any mental illness:

*“I do not think I suffer from schizophrenia. I am completely normal, and I do not have the other syndrome neither… I was hospitalized because I made an error.”(participant A9).*

Eight participants preferred a label of salience syndrome, five of schizophrenia, two preferred a mix of both labels and four rejected both labels (“I don’t want any of them”). There was a significant link between self-reported diagnoses and preferred labels (χ^2^ (9) = 24.75,p<0.005). Indeed, all those preferring a label of schizophrenia (N = 4) had also initially self-identified with a diagnosis of schizophrenia. A first reason mentioned to prefer a label of schizophrenia was perceived good fit with their personal experiences: “This one fits my case better” B9, “I have not experienced a salience syndrome, but schizophrenia yes” B5, “I: Why is [schizophrenia] describing you better? A: I hear voices” B6, “schizophrenia – this reminds me a little bit of myself” A8. With further questioning, we also discovered that this preference for schizophrenia was the result of a painful acceptance process and that the participants were not ready to dispose of the explanatory power of this diagnostic label in favor of an unknown and obscure new label: “the salience syndrome is less clear for me than schizophrenia, which I know what it is, I think it is that neurotransmitters are too active. […] When you understand that voices are hallucinations, it is over. You can then still experience voices, but you know it’s not serious” B6. Another participant expressed a feeling of relief related to receiving a diagnosis: “I think that having received the diagnosis of schizophrenia has helped me because I now understand the symptoms better, as well as what is true or what is not that true. I understand how this disease affects me” A8.

The choice of the salience syndrome label was not clearly fitting with their subjective experiences. In fact, most found the salience syndrome concept and the respective clinical vignette obscure, in spite of additional explanations by the interviewers:

“Salience syndrome, it’s something that is less, I don’t know, I didn’t know it, I’m not sure what it means really’’. Moreover, none of the initial illness narratives could be identified as similar to the salience syndrome descriptions offered in the scientific literature. One participant was unsatisfied with the two choices: “None… do you only have two choices?”, while another rightly remarked that « it is never fun to receive a diagnosis» and then refused to endorse any of the two labels. Only one participant endorsed the salience syndrome label while saying enthusiastically that this was a very good description of his illness experience.

Questioned about the stereotypes evoked by the two labels, 17 of the 18 participants reported having witnessed negative stereotypes of schizophrenia, with violence and danger (14 participants) pernicious in mass media “In the medias, each time they talk about schizophrenia, it is when there is a murder. Like what happened in Arizona last week. […] This is why I do not want to be associated with this [label of schizophrenia]”A8 and “I think it is the word. In general, people think that schizophrenia is very severe, that people are violent or aggressive”. The advantage of the salience syndrome resided in its novelty and the related lack of attached stereotypes.

Unsurprisingly, the affective reactions reported or expected for schizophrenia were those of fear and sadness: “ the diagnosis of schizophrenia, the psychiatrist put it on a paper, he did not tell it in person. Luckily, because I would have cried in front of him. But when I saw it on the welfare certificate, I cried, I went to the washroom, in the hospital, and I cried a lot. I didn’t like it” A3. In contrast, salience syndrome evoked only neutral reactions and, in two cases, curiosity.

The capacity to normalize symptoms and to make them understandable by the entourage were reported by three participants as an advantage of the salience syndrome description: “a lot of people can identify with these” B2, “I would feel comforted… because the diagnosis would make a bit more sense, people could understand it” and “well schizophrenia (…) for my family, it would affect them more because they couldn’t understand it, they can’t hear voices, the hallucinations”. Moreover, most participants feared rejection by their peers in cases of diagnoses of schizophrenia. The rejection was feared from distant friends and people in the workplace, while family was generally described as supportive and non-stigmatizing.

When we explored the reasons for preferring one label over the other, the potential for concealment was repeatedly evoked:

“(…) I would like to have another name that I could use when I will be back in society, so I could tell the truth, but they won’t really understand it. I don’t want to lie so I think I’ll just say I have the salience syndrome, yep, that’s it’ “.

One participant proposed a multiplication of synonyms for schizophrenia, such that each person could invent her own illness label and could conceal it from the rest of the society:

“I am convinced that if we changed the word more often, if there were a thousand different synonyms for schizophrenia, it would help patients. Sometimes it’s this word, sometimes this other, or this other, so people in society wouldn’t really know. Because [schizophrenia] is not well perceived.”

While the power of words and of stereotypes was acknowledged, often reasons for preferring one label over the other were more pragmatically motivated. Pragmatic reasons included: 1) receiving less medication (“As long as they don’t give me too many pills. (laugh)”), 2) prognosis (“Well, because of the [diagnosis of salience syndrome], she didn’t adjust well to life at school”) and 3) social acceptance of symptoms (“…because no one wants to live with someone who hears voices”; “… for the salience syndrome, my family would be more open-minded about it.”).

There were no significant differences in terms of self-stigma (rated on the ISMIS) between individuals when we consider the four diagnostic preferences (preferring schizophrenia, preferring salience, preferring a mix, or refusing all diagnoses). However, when we compared those who preferred the diagnosis of schizophrenia to those preferring the salience syndrome, there was a trend for lower self-stigma on the withdrawal scale in the latter group (t(11) = 2.05, p = 0.08, M = 1.62 vs M = 2.0). Given the small N and the lack of power here (and the multiple uncorrected comparisons), this result should be considered with caution, as exploratory.

## Discussion

Contrary to results from Japan [9], in the undergraduate population of study 1, the label used did not elicit different attitudes regarding people with schizophrenia or salience syndrome. As seen in other studies, the students held stigmatizing attitudes regarding social distance in work and social situations. The attitudes appeared based on the vignette and description (prescription of antipsychotic medication might have influenced their answers), rather than the label itself. Only for psychology students did we find a slightly more positive attitude when presented with the salience syndrome compared to biology students, suggesting that prior (negative) knowledge of schizophrenia might have influenced their answers.

In discussing the name change of schizophrenia with individuals in early phases of treatment, the reasons to prefer one name over the other was mostly related to the capacity of the label to avoid societal stigma, either by completely rejecting any diagnosis, or by being able to conceal the mental illness under an obscure term. The “obscurity” of the salience syndrome was thus not a matter of concern, but a highly prized quality for some.

Because terms tend to become less obscure with time, one participant proposed even a system where new names for schizophrenia would be continuously invented. While scientists and clinicians consider negatively the lack of clarity, it was a positive, yet ephemeral, characteristic of the new label from the participant’s perspectives. Secrecy is the main lay strategy for avoiding social stigma [18], thus the positive appraisal of the obscurity of a new label by the participants in the early psychosis clinic. The negative stereotypes, separation, power imbalance and discrimination do not wane simply by changing a label and it is probable that they infuse back into the new labels as society gets to understand their link with the previous labels.

Being able to share one’s symptoms and experiences and to be understood without being rejected by others was another reason to prefer a specific label. The universal qualities of the salience processes, while not clearly resonating with the subjective experience of the participants, were considered as potentially promoting empathy and tolerance from others in society.

Participants tended to reason not in abstract terms (such as diagnostic categories), but in a more concrete, pragmatic and direct manner. They focused more on symptoms, interpersonal implications and prognostic values of each diagnosis. Although the sample size is too small to extrapolate, there was a trend toward individuals preferring salience syndrome over schizophrenia as having less self-stigmatizing attitudes of withdrawal. Future studies with larger samples are warranted in order to clarify the role of labels on self-stigmatizing attitudes.

Our results somewhat differ from Kingdon et al’s exploratory study, where multiple terms were proposed as an alternative to schizophrenia [19], affirming that « *it may be that stigmatisation arises from the nature of the illness as opposed to its terminology but the current term is semantically inexact–essentially meaningless–and increasingly associated inappropriately with violence and deterioration*. » They explored the attitudes of 27 patients, care coordinators and consultant psychiatrists to the term « schizophrenia » and to four newly proposed psychosocial alternatives (sensitivity psychosis, drug-related psychosis, traumatic psychosis, anxiety psychosis). Overall, 74% of the patients preferred one of the new terms over schizophrenia and most of them held positive or neutral attitudes towards the new terms. Interestingly, while care coordinators shared patients’ preference for new terms, psychiatrists were more negative, fearing the imprecision of the descriptions or the mixing of nosological categories (anxiety and psychosis). There was little agreement between patients, psychiatrists and care coordinators regarding the best term for each patient.

Our study has some limitations. Study 1 only included undergraduate students, a fairly educated sample, and might not represent the general population. Study 2 was conducted with a fairly small convenience sample of individuals, who were all quite verbal and all mentioned being supported by their families, which is not always the case in this clinical population. Inter-rater consensus of qualitative scores was not obtained, although the interviewers confirmed the qualitative analyses. Only one alternative to the term schizophrenia was proposed; perhaps more choices would have generated different results. And, of course, this study did not explore the scientific or clinical validity of these nosological entities.

In conclusion, our results do not support Kapur (2003) and van Os (2009)’s hypotheses regarding 1) utility of the salience syndrome for explaining the subjective experiences of patients and 2) sustained de-stigmatizing effects. Most participants preferred the salience syndrome label because of its novelty and “obscurity” as this would help them avoid stigma. However, in the event of a broader adoption, many believed that the “salience syndrome” would likely become stigmatizing. For those suffering from mental illness, a name change for schizophrenia to “salience syndrome” might thus offer only a temporary relief from stigma. This highlights the importance of using sound scientific models of stigma and empirical validations when claiming de-stigmatizing effects of an intervention, such as changing the name of schizophrenia. Similar studies could inform the debates over nosological shifts, such as those observed during the DSM-V preparation.

## Supporting Information

Table S1 Mean scores (and standard deviations) for each question for each version of the survey of Study 1 (0 is neutral, positive scores “likely”, negative scores “unlikely”).
(DOCX)

Appendix S1 Clinical vignette example.
(DOCX)

## References

[1] Bleuler E (1908) Die Prognose der Dementia praecox (Schizophreniegruppe). Allgemeine Zeitschrift fur Psychiatrie 65: 436.

[2] Lieberman JA, First MB (2007) Renaming schizophrenia. BMJ 334: 108. 17235058 10.1136/bmj.39057.662373.80 PMC1779873

[3] Kingdon DG, Kinoshita Y, Naeem F, Swelam M, Hansen L, et al (2007) Schizophrenia can and should be renamed. BMJ 334: 221–222. 10.1136/bmj.39108.396852.1F PMC1790791 17272533

[4] (2007) Renaming schizophrenia editorial - rapid responses; BMJ 334: 108. Available: http://www.bmj.com/content/334/7585/108?tab=responses. Accessed 2012 Dec 4.

[5] Link BG, Yang LH, Phelan JC, Collins PY (2004) Measuring mental illness stigma. Schizophr Bull 30: 511–541. 15631243 10.1093/oxfordjournals.schbul.a007098

[6] First MB (2006) Deconstructing Psychosis. Available: http://www.dsm5.org/Research/Pages/DeconstructingPsychosis(February15-17,2006).aspx. Accessed 2012 Dec 4.

[7] APA (2000) Diagnostic and statistical manual of mental disorders : DSM-IV TR. Washington: American Psychiatric Association.

[8] George B (2010) What’s in a name? Client participation, diagnosis and the DSM-5. Journal of mental health (Abingdon, England) 19: 479. 10.3109/09638237.2010.526157 21121820

[9] Sato M (2006) Renaming schizophrenia: a Japanese perspective. World Psychiatry 5: 53–55. 16757998 PMC1472254

[10] Takahashi H, Ideno T, Okubo S, Matsui H, Takemura K, et al (2009) Impact of changing the Japanese term for “schizophrenia” for reasons of stereotypical beliefs of schizophrenia in Japanese youth. Schizophr Res 112: 149–152. 19398303 10.1016/j.schres.2009.03.037

[11] Kingdon D, Vincent S, Vincent S, Kinoshita Y, Turkington D (2008) Destigmatising schizophrenia: does changing terminology reduce negative attitudes? Psychiatric Bulletin 32: 419–422.

[12] Corrigan PW, O’Shaughnessy JR (2007) Changing mental illness stigma as it exists in the real world. Australian Psychologist 42: 90–97.

[13] Kapur S, Mizrahi R, Li M (2005) From dopamine to salience to psychosis–linking biology, pharmacology and phenomenology of psychosis. Schizophr Res 79: 59–68. 16005191 10.1016/j.schres.2005.01.003

[14] Kapur S (2003) Psychosis as a state of aberrant salience: a framework linking biology, phenomenology, and pharmacology in schizophrenia. Am J Psychiatry 160: 13–23. 12505794 10.1176/appi.ajp.160.1.13

[15] van Os J (2009) ‘Salience syndrome’ replaces ‘schizophrenia’ in DSM-V and ICD-11: psychiatry’s evidence-based entry into the 21st century? Acta Psychiatr Scand 120: 363–372. 19807717 10.1111/j.1600-0447.2009.01456.x

[16] Boyd Ritsher J, Otilingam PG, Grajales M (2003) Internalized stigma of mental illness: psychometric properties of a new measure. Psychiatry Research 121: 31–49. 14572622 10.1016/j.psychres.2003.08.008

[17] Link BG, Phelan JC (2001) Conceptualizing Stigma. Annual Review of Sociology 27: 363–385.

[18] Goffman E (1963) Stigma; notes on the management of spoiled identity. Englewood Cliffs, N.J.,: Prentice-Hall. 147 p.

[19] Kingdon D, Gibson A, Kinoshita Y, Turkington D, Rathod S, et al (2008) Acceptable terminology and subgroups in schizophrenia. Social Psychiatry and Psychiatric Epidemiology 43: 239–243. 18196187 10.1007/s00127-007-0284-y

