# Supplementary material for: Changing the Name of Schizophrenia: Patient Perspectives and Implications for DSM-V
Source: PLoS One. 2013 Feb 14;8(2):e55998. doi: 10.1371/journal.pone.0055998 (PMC3573057; doi:10.1371/journal.pone.0055998)
Supplement: Table S1 — Mean scores (and standard deviations) for each question for each version of the survey of Study 1 (0 is neutral, positive scores “likely”, negative scores “unlikely”). (DOCX) [file pone.0055998.s001.docx]

TABLE S1

| Table S1. Mean scores (and standard deviations) for each question for each version of the survey of Study 1 (0 is neutral, positive scores “likely”, negative scores “unlikely”) | | | | | |
| --- | --- | --- | --- | --- | --- |
|  | VERSION | |  |  |  |
|  | A (salience) | B (schizophrenia) | t-test | p-value |  |
| Question 1 | -0,38 (1,02) | -0,34 (1,11) | -0,23 | 0,82 |  |
| Question 2 | 0,38 (0,96) | 0,28 (1,20) | 0,58 | 0,57 |  |
| Question 3 | 0,72 (0,86) | 0,65 (1,00) | 0,51 | 0,61 |  |
| Question 4 | -0,34 (0,93) | -0,34 (1,08) | 0,00 | 1,00 |  |
| Question 5 | -0,05 (0,99) | -0,20 (1,16) | 0,85 | 0,40 |  |
| Total score | 0,33 (2,85) | 0,05 (3,88) | 0,52 | 0,60 |  |
