# Supplementary material for: Changing the Name of Schizophrenia: Patient Perspectives and Implications for DSM-V
Source: PLoS One. 2013 Feb 14;8(2):e55998. doi: 10.1371/journal.pone.0055998 (PMC3573057; doi:10.1371/journal.pone.0055998)
Supplement: Appendix S1 — Clinical vignette example. (DOCX) [file pone.0055998.s002.docx]

Appendix S1: Clinical vignette example

**Schizophrenia**

Fred, a college student is brought to the hospital by a friend for an evaluation. Fred was initially doing well in school. In the last few months, his friends noticed that he had become more isolated and distracted. They also noticed that he missed school for the last six weeks. During the psychiatric examination, Fred seemed disheveled, distracted, and had difficulties expressing himself. He seemed guarded, and after further questioning, he mentioned believing that his grades had gone down because his teachers targeted him, ‘disliked’ him, and that he had heard them speak in those terms.

Fred is given the diagnosis of ‘schizophrenia’ since he has been presenting with delusions and potentially auditory hallucinations for more than a month.

**Salience syndrome**

Steve, a college student, is brought to the hospital by a friend for an evaluation. Steve was initially doing well in school. However, overtime, he has become distracted by minor events and seems to get lost in details. His friends often found that he lacked common sense in his conversations and behaviours. During the psychiatric examination, Steve had difficulties understanding the context of the evaluation and often became lost in his reflections that were not related to the situation. He described experiencing the world in a new way. He mentioned understanding things that others couldn’t. His surroundings and society in general, according to him, were not open to his discoveries, which often caused conflicts. He therefore decided to withdraw from social activities and from school.

Steve is given the diagnosis of ‘salience syndrome’, meaning that he gives too much attention and importance to internal and external stimuli, and that this increased attention brings about unusual experiences and beliefs not held by his surroundings.
